# PVDF HFP_RuO_2_ Nanocomposite Aerogels Produced by Supercritical Drying for Electrochemical Oxidation of Model Tannery Wastewaters

**DOI:** 10.3390/nano11061436

**Published:** 2021-05-29

**Authors:** Maria Sarno, Carmela Scudieri, Eleonora Ponticorvo, Lucia Baldino, Stefano Cardea, Ernesto Reverchon

**Affiliations:** 1 Department of Physics “E.R. Caianiello”, University of Salerno, 84084 Fisciano, SA, Italy; 2 NANO_MATES, Research Centre for Nanomaterials and Nanotechnology at the University of Salerno, University of Salerno, 84084 Fisciano, SA, Italy; linascudieri@yahoo.it (C.S.); eponticorvo@unisa.it (E.P.); 3 Department of Industrial Engineering, University of Salerno, 84084 Fisciano, SA, Italy; scardea@unisa.it (S.C.); ereverchon@unisa.it (E.R.)

**Keywords:** RuO_2_ nanoparticles, PVDF HFP aerogel, supercritical CO_2_ drying, electrochemical oxidation, tannery wastewater

## Abstract

A supercritical CO_2_ drying process was used to prepare an innovative nanocomposite, formed by a poly(vinylidene fluoride-co-hexafluoropropylene) (PVDF HFP) aerogel loaded with RuO_2_ nanoparticles. The produced nanocomposites, at 10% and 60% *w*/*w* of RuO_2_, were tested for the electrochemical oxidation of model tannery wastewaters. The effect of the electrochemical oxidation parameters, like pH, temperature, and current density, on tannic acid, intermediates, and chemical oxygen demand (COD) removal, was investigated. In particular, the electrolysis of a simulated real tannery wastewater, using PVDF HFP_RuO_2_ 60, was optimized working at pH 10, 40 °C, and setting the current density at 600 A/m^2^. Operating in this way, surfactants, sulfides, and tannins oxidation was achieved in about 2.5 h, ammonium nitrogen oxidation in 3 h, and COD removal in 5 h. When chloride-containing solutions were tested, the purification was due to indirect electrolysis, related to surface redox reactions generating active chlorine. Moreover, sulfide ions were converted into sulfates and ammonium nitrogen in gaseous N_2_.

## 1. Introduction

Electrochemical oxidation (EO) is an environmentally compatible technique that uses electrons to oxidize pollutants into carbon dioxide and water, or other oxides, without generating secondary pollutants [1,2]. According to Kim et al. [3], EO can be improved using composite materials organized at nanoscale, since they increase the density of electrodes surface active sites.

Poly(vinylidene fluoride-co-hexafluoropropylene) (PVDF HFP) aerogels, containing ruthenium oxide (RuO_2_) nanoparticles (NPs), can be outstanding candidates for electrochemical reactions. In particular, PVDF HFP is characterized by a good electrochemical stability and affinity to polar liquid electrolytes [4,5,6]; whereas RuO_2_ is widely active in redox reactions [3,7,8,9]. These properties, coupled with the possibility to potentiate the electrochemical oxidation activity using aerogels organized in a hierarchical structure from micro- to nanoscale [10], open perspectives for an industrial application of these nanocomposite systems. Indeed, polymeric nanocomposites can be considered as a supporting complementary purification technology for wastewaters treatment [11], since the traditional physicochemical primary (precipitation, filtration, …) and secondary (microbiological) processes, using chemicals and acid treatments, are not always effective in achieving pollutants residue below the admitted threshold value [12,13,14]. Due to their EO capacity, these advanced systems instead (i) can treat wastes in the liquid and in the solid phase through electro-assisted oxidation, reduction, and deposition, or flotation and coagulation; (ii) can be easily automated through the acquisition of current and potential signals; and (iii) the addition of toxic chemical agents is not required.

This challenge is mostly related to highly complex wastewater mixtures, as in the case of the vegetable tannery industries. These industries use large amounts of chemicals (i.e., salts, tannin agents, surfactants, NH_3_, sulfides, chlorides, etc.) for the treatment of skins [12,13] that are difficult to be disposed safely. The organic pollutant anodic oxidation can occur through direct electron transfer and indirect oxidation mediated by reactive intermediates, such as hydroxyl radical (OH radical) and reactive chlorine species [15].

Among the active materials for anodic oxidation, RuO_2_, IrO_2_, Pt, show low oxygen evolution reaction (OER) overpotential, can mineralize pollutants in the presence of chlorine species or strongly physisorb OH radicals. Some authors tried to use a RuO_2_- based electrode to treat tannery wastewaters. Bai et al. [16] prepared, via thermal decomposition, a Ti/SnO_2_-RuO_2_ electrode, on which electrocatalytic degradation of bromocresol green (BCG) was tested. The removal efficiency of BCG on the Ti/SnO_2_-RuO_2_ electrode was determined in terms of chemical oxygen demand (COD) and ultraviolet-visible absorption spectrometry. At the optimal conditions (initial concentration of 100 mg L^−1^, pH 7, reaction temperature of 30 °C, current density of 12 mA cm^−2^ and electrolysis time of 150 min), these authors obtained a removal efficiency of BCG up to 91%. Fajardo et al. [17] applied an EO process using Ti/RuO_2_ anodes to phenolic wastewaters. Working at 10 g L^−1^ of NaCl, 119 mA cm^−2^ and initial pH of 3.4, a complete removal of the total phenolic content and COD in the phenolic mixture tested was obtained. Kaur et al. [18] used a RuO_2_-coated Ti electrode (Ti/RuO_2_) for the EO treatment of textile wastewater. They demonstrated that most of the organics was completely eliminated during EO process. Chauhan et al. [19] used Ti/RuO_2_ and Fe as anode and cathode, respectively, for the treatment of wastewater containing a high concentration of nitrate ion. The maximum NO^3−^ reduction efficiency of ≈46% was obtained at J = 214.29 A m^−2^ after 180 min. A maximum total nitrogen removal efficiency of ≈ 51% was obtained at pH 12 and J = 285.71 A m^−2^.

Supercritical CO_2_ (SC-CO_2_)-assisted drying is emerging as a green alternative to the corresponding traditional processes (e.g., freeze drying, air drying, etc.) for the production of aerogels, thanks to the properties of supercritical fluids: mainly, gas-like diffusivity, liquid-like density, and near to zero surface tension. These features allow to obtain polymeric devices characterized by a regular and homogeneous nanostructure, with open and interconnected pores [20,21]. Moreover, in the case of aerogels loaded with bioactive compounds, a homogeneous distribution is assured thanks to the fast solvent removal [22,23]. Negligible toxic organic solvent residues in the final product are also obtained, making these materials safe for various applications. In a previous paper, Sarno et al. [10] reported the use of SC-CO_2_ assisted drying for the preparation, in a one-step, of a novel porous device loaded with molybdenum disulfide (MoS_2_) nanosheets. It consisted of three layers: the middle layer was formed by PVDF HFP aerogel alone; whereas the top and bottom layers were formed by PVDF HFP aerogels containing dispersed MoS_2_. This device was tested as a supercapacitor and, at the optimized operative conditions, it achieved an excellent specific capacitance of 176 F/g and a very high energy density of 97.8 Wh/kg, at a power density of 0.65 kW/kg (current density 0.6 A/g).

Therefore, this paper is aimed at producing, via supercritical CO_2_ drying, an innovative nanocomposite system formed by a PVDF HFP aerogel loaded with RuO_2_ NPs, to be tested for the electrochemical oxidation of model tannery wastewaters. The produced nanocomposites will be characterized by various physicochemical techniques, and the effect of EO parameters, like pH, temperature and current density, on tannic acid, intermediates, and COD removal, will be investigated. For the first time, the advantage of using polymeric aerogels, structured from micro- to nanoscale, is joined together the nanomaterials’ properties, to improve the final purification performance of a simulated real tannery wastewater mixture.

## 2. Materials and Methods

Polyvinylidene fluoride hexafluoropropylene (PVDF HFP, density 1.77 g cm^−3^, average M_w_ ~400,000) was supplied by Solvay S.A. (Ixelles, Belgium); acetone (99.5% purity, Sigma Aldrich, Milan, Italy) was used as solvent, and ethanol (99.8% purity, Sigma Aldrich, Milan, Italy) as non-solvent, for gel formation. CO_2_ (99% purity) was purchased from Morlando Group (Sant’Antimo, Naples, Italy). RuCl_3_·3H_2_O, NaOH (ACS reagent, ≥97.0%), Na_2_SO_4_ (ACS reagent, ≥99.0%), tannic acid (ACS reagent), NaCl (purity ≥ 99.5%), and NH_4_Cl (purity ≥ 99.5%), were purchased from Sigma Aldrich (Milan, Italy).

### 2.1. RuO_2_ Nanoparticle Synthesis

RuO_2_ nanoparticles were synthesized using RuCl_3_·3H_2_O and NaOH. In particular, 200 mg of RuCl_3_·3H_2_O were added to 120 mL of distilled water; pH was adjusted to 7 using NaOH (0.1 M). The mixture was heated for 6 h in reflux at 110 °C [24]. After the reaction, the precipitate was separated, dried, and treated for 6 h at 150 °C. In order to obtain the formation of nano-oxides, the recovered powder was calcined at 400 °C. Specifically, three 1 h steps followed: (I) between room temperature and 250 °C; (II) up to 350 °C; and, finally, (III) to 400 °C, at a heating rate of 2 °C/min, obtaining RuO_2_ NPs (in the following RuO_2_) [25].

### 2.2. Aerogel Preparation Procedure

PVDF HFP aerogels were obtained starting from two different systems: the first one containing pure PVDF HFP; whereas the second one formed by RuO_2_-loaded PVDF HFP.

PVDF HFP powder, at 10% *w*/*w*, was dissolved in acetone (60% *w*/*w*) and, subsequently, ethanol, at 30% *w*/*w*, was added dropwise to the homogeneous solution (i.e., PVDF HFP/acetone). The obtained sample was frozen at −25 °C for 2 h to favour gel (PVDF HFP/acetone/ethanol) formation.

In the second system, PVDF HFP solutions were loaded, respectively, with RuO_2_ at 10% and 60% *w*/*w* with respect to the polymer weight (10% *w*/*w*). A nanoparticle loading larger than 60% *w*/*w* was not tried since a previous study [23] demonstrated that aerogel nanoporosity tended to decrease due to nanopores occlusion and structure saturation at nanometric level due to the nanoparticles excess. RuO_2_ was sonicated for 10 min in 1 mL of acetone, using a tip sonicator, to improve particle dispersion in the liquid medium. This suspension was added to the PVDF HFP/acetone solution, previously prepared. The final system was sonicated for 10 min in a sonicator bath, at room temperature. Then, as in the case of pure PVDF HFP, ethanol, at 30% *w*/*w*, was added to the homogeneous suspension (i.e., PVDF HFP/acetone/RuO_2_), and the sample was frozen at −25 °C for 2 h, until the loaded physical gel (PVDF HFP/acetone/ethanol/RuO_2_) was formed.

Aerogels were produced using a laboratory setup [23] constituted by a 316 stainless steel cylindrical vessel, with an internal volume of 200 mL. Gels (about 15 mm diameter and 2 mm height) were placed in the vessel that was closed and filled with CO_2_ up to the desired pressure, using a high-pressure pump (mod. LDB1, Lewa, Leonberg, Germany). Pressure in the vessel was measured by a test gauge (mod. MP1, OMET, Lecco, Italy) and regulated using a micrometering valve (mod. 1335G4Y, Hoke, Spartanburg, SC, USA). Temperature was regulated using PID controllers (mod. 305, Watlow, Corsico (MI), Italy). At the exit of the vessel, a rotameter (mod. d6, ASA, Sesto San Giovanni (MI), Italy) was used to measure CO_2_ flow rate. At the end of the experiment, the system was slowly depressurized and samples were collected.

### 2.3. Characterizations

Morphological and chemico-physical characteristics of the nanocomposite aerogels were investigated by different techniques.

A Tecnai electron microscope (mod. Tecnai G2, 20 S-Twin, FEI, Hillsboro, OR, USA), operated at 200 kV with a LaB6 filament, as the source of electrons, was used for the transmission electron microscopy (TEM) analysis.

Samples were cryo-fractured using liquid nitrogen (SOL, Milan, Italy); then, they were sputter coated with gold (Agar Auto Sputter Coater mod. 108 A, Stansted, UK) at 30 mA for 100 s, and analyzed by a field emission scanning electron microscope (FE-SEM, mod. LEO 1525, Carl Zeiss SMT AG, Oberkochen, Germany) to observe their morphology.

Samples were cryo-fractured using liquid nitrogen and sputter coated with chromium (Peltier cooled K575X, EMITECH, Ashford, Kent, UK); then, they were analyzed by energy dispersive X-ray spectroscopy (EDX INCA Energy 350, Oxford Instruments, Gometz la Ville, France) to determine the dispersion of RuO_2_ in the PVDF HFP matrix: Ru atoms were selected for RuO_2_ and C atoms for PVDF HFP.

XRD analyses were performed using a D8 X-ray diffractometer (Bruker, Macerata, Italy) with a CuKα radiation source.

Thermogravimetric analysis (TG-DTG), at a 10 °C/min heating rate in flowing air, was performed using a SDTQ 600 Analyzer (TA Instruments, New Castle, DE, USA).

Raman spectra were obtained at room temperature using a microRaman spectrometer (inVia, Renishaw, Pianezza, TO, Italy, 514 nm excitation wavelength, laser power 30 mW).

In order to measure aerogels porosity, the *n*-butanol adsorption method was adopted, as reported in the literature [23,26].

N_2_ adsorption-desorption isotherms (BET surface area) of the RuO_2_ based-electrode were measured at −196 °C by a 1042 V3.12 system (COSTECH Instruments, Cernusco S/Nav, MI, Italy) after a pretreatment of the electrode performed at 250 °C for 3 h, in a helium environment. Nanocomposite aerogels are named in the following discussion as PVDF HFP_RuO_2_ 10 and PVDF HFP_RuO_2_ 60.

### 2.4. Tannery Wastewater Treatment

A model tannery wastewater solution was prepared dissolving 9 g/L of Na_2_SO_4_, 1.6 g/L of tannic acid, 3 g/L of NH_4_Cl, 6 g/L of NaCl, 0.08 g/L of surfactants, and 0.08 g/L of H_2_S (RPE reagent, Carlo Erba, Cornaredo, MI, Italy) in distilled water. This model solution simulated a real tannery wastewater after biological oxidation. A simpler solution was also prepared, containing tannic acid, NaCl and Na_2_SO_4_.

A galvanostatic flow cell was used for electrolysis. A platinum square electrode of 5 cm^2^ was adopted as the cathode. The working electrode was formed by PVDF HFP_RuO_2_ nanocomposite and exhibited the same size of 5 cm^2^ (interelectrode gap of 0.2 cm). A thermoregulated glass cylinder was used for the solution; a peristaltic pump allowed a flux of 200 L/h. NaOH or H_2_SO_4_ were added to adjust pH.

Absorbances at 420 and 280 nm were determined using a V-570 spectrophotometer (Jasco, Easton, MD, USA) for color and tannic acid determination, respectively. A LASA50 system (DR LANGE, Düsseldorf, Germany) was used to determine chemical oxygen demand (COD) and amounts of surfactants, ammonium, sulfides, and nitrates. The yield, expressed in terms of space-time yield (STY), was used to quantify the efficiency of the electrochemical oxidation [27]:

STY = (CE M A i)/(n F) × 3600

where: CE is the current efficiency; M is the molar weight, in g/mol; A is equal to the ratio between area and volume of the electrode, in m^−1^; i is the current density, in A/m^2^; n is the number of electrons involved in the reaction; F the Faraday constant. CE, for the anodic oxidation of the organic compounds, was calculated from [27]:

CE = (COD_t_ − COD_t+∆t_)/(8I_∆t_) × (F V)

with COD at time t and t + ∆t, in g_O2_/L; I the current, in A; and V the electrolyte volume, in L.

## 3. Results and Discussion

### 3.1. RuO_2_ Nanoparticle Characterization

**Figure 1:**
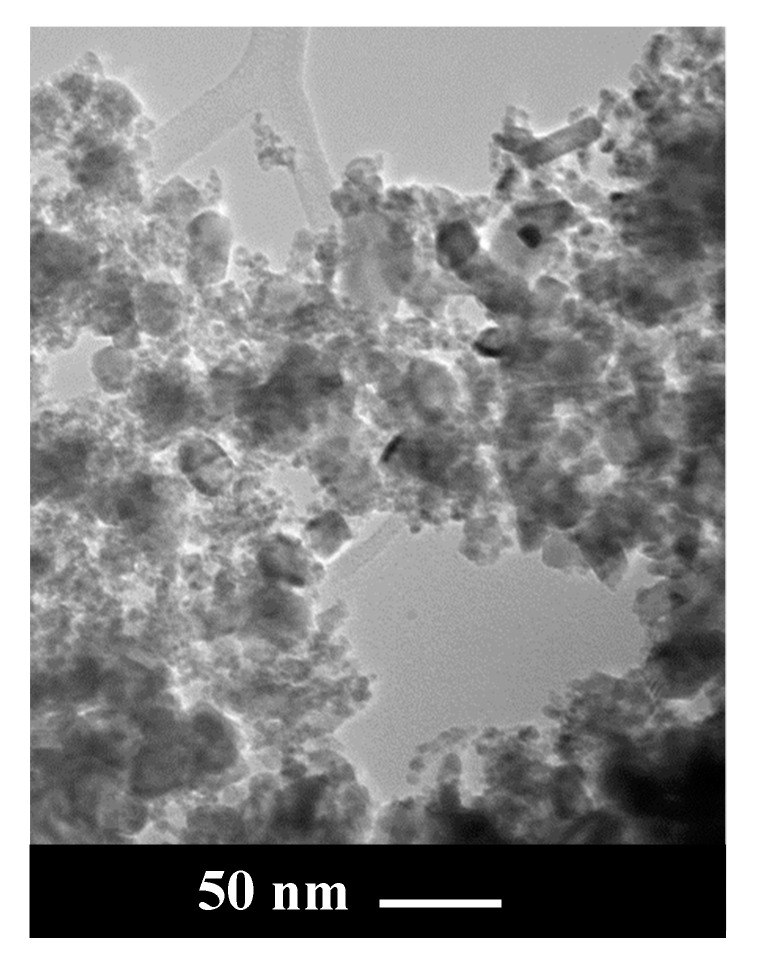
TEM image of RuO_2_.

RuO_2_ powder morphology was analyzed by TEM. As illustrated in Figure 1, nanoparticles of different size were observed in the sample: in particular, larger nanoparticles, with size in the range 20–30 nm, and a lot of smaller NPs with diameters in the range 3–4 nm, were also present.

**Figure 2:**
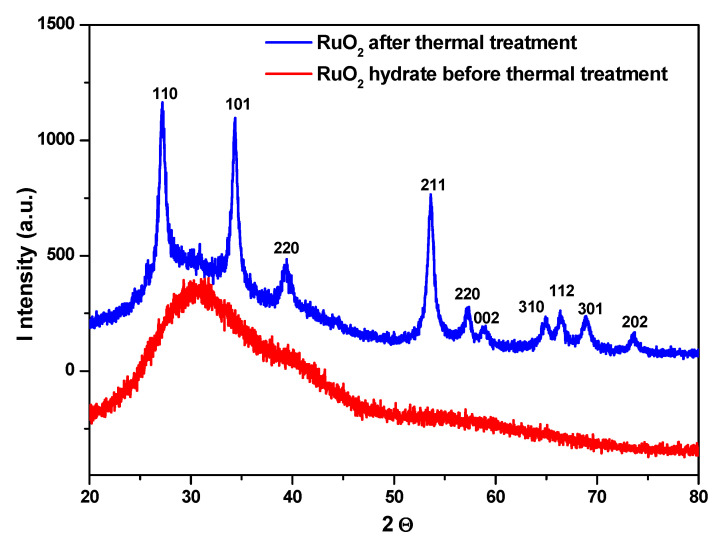
XRD pattern of RuO_2_, before (red line) and after (blue line) thermal treatment.

RuO_2_ was analyzed before and after calcination by XRD, as shown in Figure 2. No diffraction peaks can be detected in the XRD pattern of RuO_2_ before calcination (red line of Figure 2); only a shoulder at around 30.6° was observed, indicating a poor crystallinity [25], likely due to powder hydration [25,28].

The typical peaks of RuO_2_ can be observed after calcination (blue line of Figure 2), which can be indexed as the rutile RuO_2_ [25,29], at 2ϑ = 27.3°, 34.3°, 39.5°, 53.6°, 57.1°, 59.0°, 65°, 66.5°, 68.9° and 73.6°, corresponding to: (1 1 0), (1 0 1), (2 0 0), (2 1 1), (2 2 0), (0 0 2), (3 1 0), (1 1 2), (3 0 1) and (2 0 2), respectively [30].

**Figure 3:**
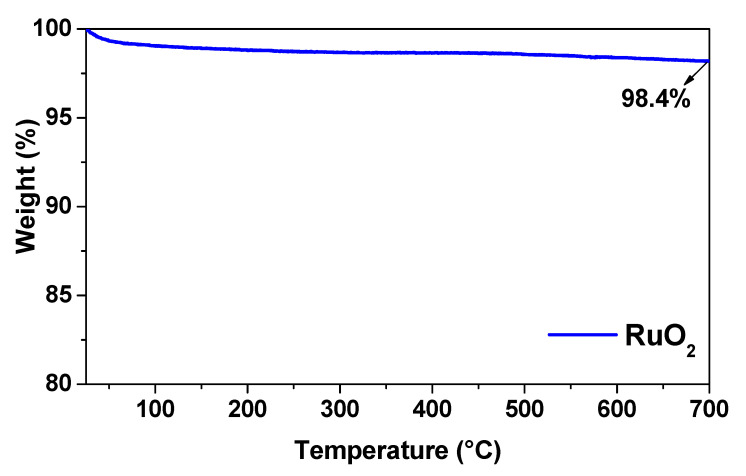
TG analysis of RuO_2_.

Thermogravimetric analysis (TGA) of RuO_2_ is reported in Figure 3. In the temperature range tested (25–700 °C), the weight loss was negligible. In particular, increasing temperature from 25 to 700 °C, the total weight loss for RuO_2_ represented less than 2% of the sample mass. These losses occurred over the entire temperature range and can be attributed to water adsorbed in the oxide particles [25].

**Figure 4:**
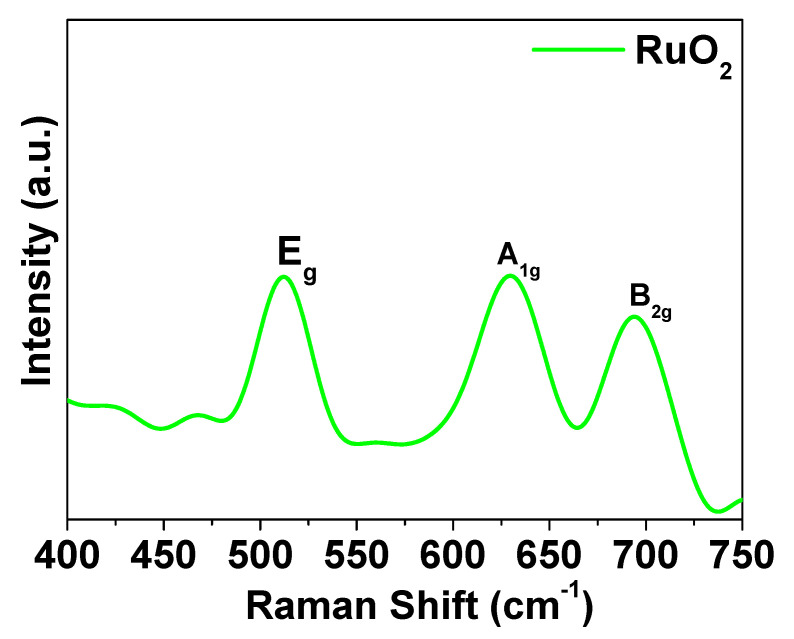
Raman spectrum of RuO_2_.

Figure 4 shows the Raman spectrum of RuO_2_ nanoparticles. The typical RuO_2_ peaks, at about 502 cm^−1^ (E_g_ band), at about 618 cm^−1^ (A_1g_ band), and at about 676 cm^−1^ (B_2g_ band), were detected [28].

### 3.2. Nanocomposite Aerogel Characterization

PVDF HFP aerogels alone and loaded with RuO_2_ were produced by supercritical CO_2_ drying at 35 °C and 200 bar (ρ_CO2_ = 0.866 g/cm^3^) for 3 h. These operative conditions were optimized in a previous work, using a similar nanocomposite system [10].

**Figure 5:**
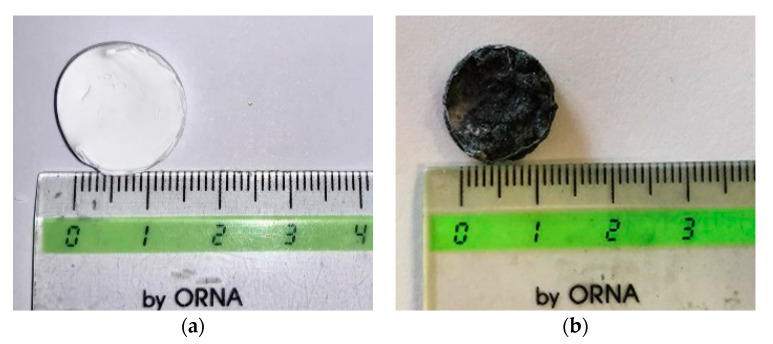
Photographs of the PVDF HFP aerogel (**a**) and of the RuO_2_ loaded PVDF HFP composite aerogel (**b**).

The first macroscopic observation was related to the samples shape and colour, as shown in Figure 5a,b. PVDF HFP aerogel (Figure 5a) preserved the starting wet gel volume (dimensions reported in Section 2.2); whereas a slight shrinkage was observed in the case of the nanocomposite (aerogel diameter of about 13 mm vs. 15 mm of the starting wet gel) reported in Figure 5b, confirming that the process operative conditions were properly selected. More specifically, supercritical drying was carried out at a negligible surface tension of the supercritical mixture (CO_2_ + acetone + ethanol) that avoided the collapse of the characteristic nanostructured gel morphology, and the consequent drastic sample volume reduction. Moreover, in the case of RuO_2_-loaded PVDF HFP gels, a uniform black colour characterized these samples, suggesting a homogeneous NPs distribution inside the polymeric matrix (see Figure 5b).

**Figure 6:**
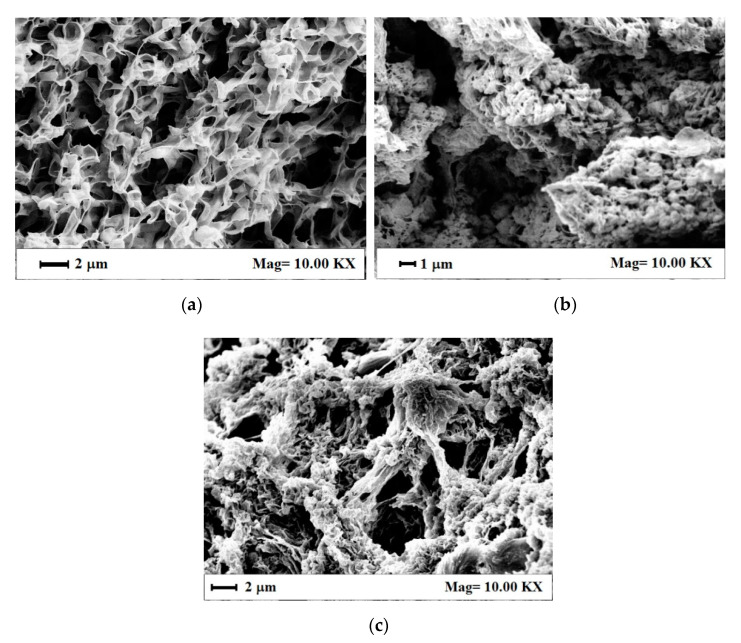
SEM images of the internal aerogel section: (**a**) PVDF HFP alone, (**b**) PVDF HFP_RuO_2_ 10, (**c**) PVDF HFP_RuO_2_ 60.

The morphological features of these aerogels were observed by SEM to determine the possible effect of RuO_2_ loading on the PVDF HFP gel formation and structure. As shown in Figure 6a, PVDF HFP aerogel alone was characterized by a regular and sub-micrometric porous structure, with interconnected pores of size lower than 1 μm, suitable to host nanoparticles. PVDF HFP_RuO_2_ nanocomposites morphology, shown in Figure 6b,c, presented some aggregates intercalated in the polymeric matrix; this grape cluster structure was more evident at the largest RuO_2_ loading in the starting suspension (see Figure 6c). An explanation of this phenomenon can be related to RuO_2_ that probably nucleated and aggregated spontaneously in the polymeric liquid system, and sonication favored a homogeneous distribution of these clusters into the sample before gel formation. This RuO_2_ dispersion was maintained after supercritical drying, thanks to the fast kinetics in solvents removal from the samples that avoided clusters deposition on the bottom of the PVDF HFP aerogels.

**Figure 7:**
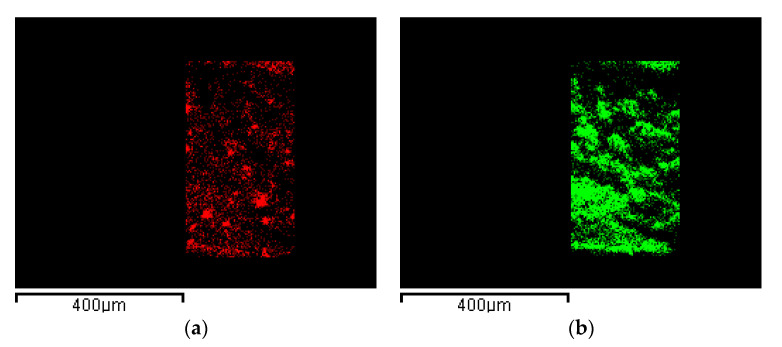
EDX maps of: (**a**) PVDF HFP network identified by C atoms and (**b**) RuO_2_ identified by Ru atoms.

This hypothesis was verified by EDX analysis, reported in Figure 7. In particular, C atoms were selected for PVDF HFP (Figure 7a) and Ru atoms for RuO_2_ (Figure 7b), as described in Characterizations. This analysis demonstrated that RuO_2_, represented in green, was uniformly dispersed in the polymeric matrix (red map). This result is relevant, since a constant nanocomposite electrochemical performance can be expected, whatever is the region of the aerogel surface exposed to the reaction.

Porosity of PVDF HFP aerogel alone and of RuO_2_ containing nanocomposites was measured as previously [23]. The results reported a PVDF HFP aerogel porosity of 78.5% that increased with RuO_2_ content from about 80%, at 10% NPs loading, up to the maximum value of 88.2%, when RuO_2_ loading was 60% *w*/*w*, in agreement with the morphological study (Figure 6c). N_2_ adsorption-desorption allowed to obtain the RuO_2_ based-electrode surface area that resulted equal to 66 m^2^/g for PVDF HFP_RuO_2_ 10 and to 61 m^2^/g for PVDF HFP_RuO_2_ 60.

**Figure 8:**
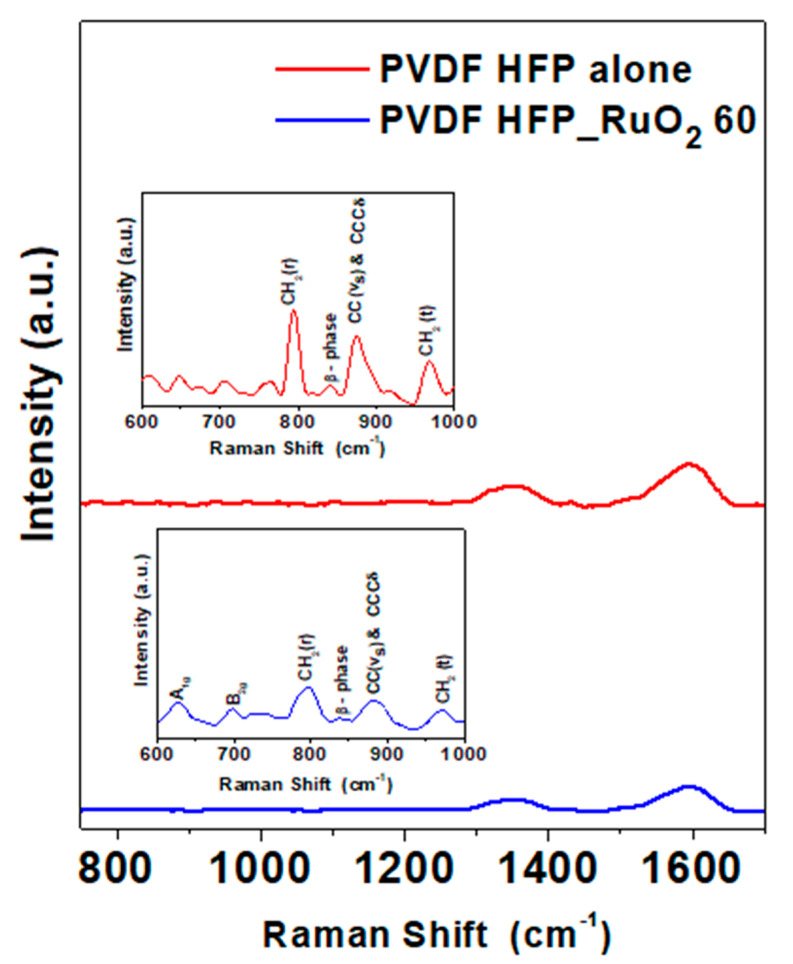
Raman spectra of PVDF HFP aerogel alone (red line) and of PVDF HFP_RuO_2_ 60 (blue line).

Raman spectra of PVDF HFP aerogels are reported in Figure 8. In the spectrum of the PVDF HFP aerogel alone, peaks at 753 cm^−1^ and 968 cm^−1^ were found. They were due to the α-phase CH_2_ rocking and twisting vibrations, respectively [31]. The combination of CCCδ skeletal bending of C(F)–C(H)–C(F) and symmetric C–C band determined the shift of the band at 873 cm^−1^ [23,31]. The presence of the β-phase was suggested by the weak band at 838 cm^−1^ [10,31]. Raman spectrum of the PVDF HFP_RuO_2_ nanocomposite indicated a crystallinity reduction of α and β phases due to the nano-additive incorporation.

### 3.3. Electrochemical Oxidation of Model Tannery Wastewaters

**Figure 9:**
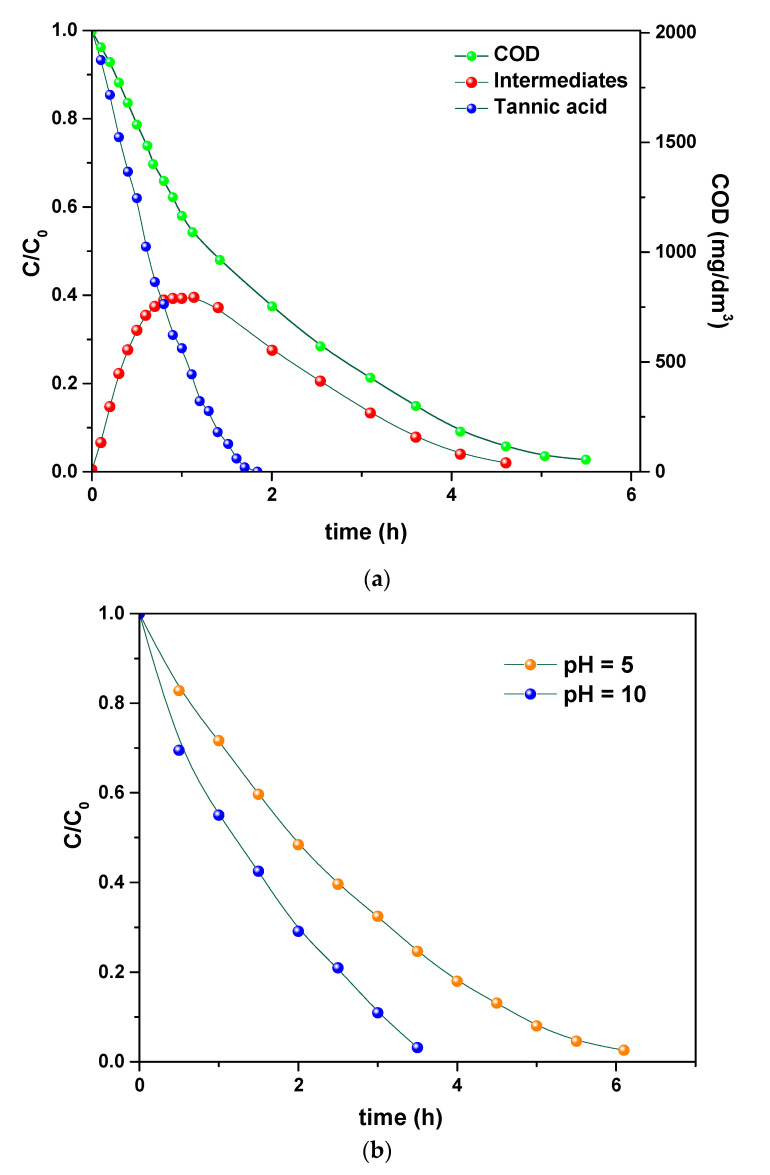

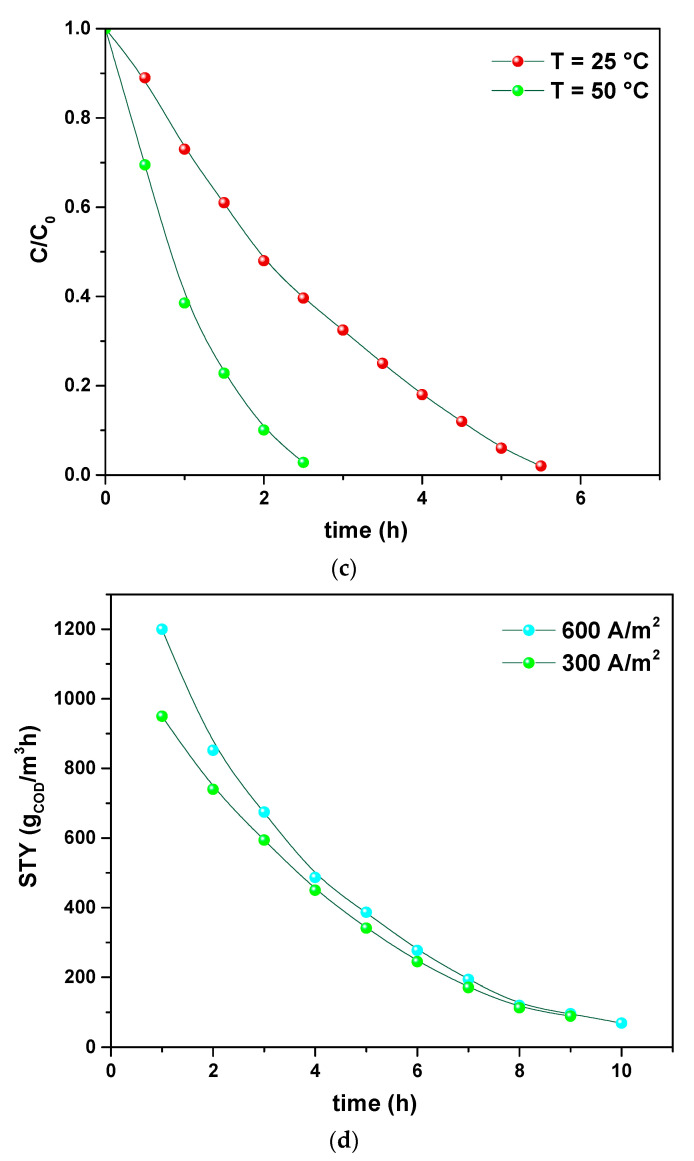
Concentrations expressed as C/C_0_ of tannic acid, organic intermediates, and COD during the simpler model tannery wastewater oxidation using PVDF HFP_RuO_2_ 60: pH = 6.5, applied current density of 600 A/m^2^, T = 20 °C (**a**). Effect of pH on COD evolution during the electrolysis of the simpler model tannery wastewater: T = 20 °C, applied current density of 400 A/m^2^ (**b**). Influence of temperature during the electrolysis of the simpler model tannery wastewater: pH = 7.5, applied current density of 450 A/m^2^ (**c**). Evolution of STY during the simpler model tannery wastewater oxidation at different applied current densities: T = 20 °C, pH = 6.5 (**d**).

Once investigated the physicochemical and morphological features of the PVDF HFP_RuO_2_ nanocomposites, they were tested as an electrode for electrochemical oxidation of a simple model tannery wastewater, containing tannic acid, NaCl and Na_2_SO_4_ and, after that, of a simulated real tannery wastewater. These model tannery wastewater solutions were prepared according to the procedure described in Section 2.4. Figure 9a–d report the results related to the treatment of the simpler model tannery wastewater, in terms of: (i) concentration of tannic acid, organic intermediates, and COD during the oxidation using PVDF HFP_RuO_2_ 60; (ii) effect of pH on COD evolution during the electrolysis; (iii) influence of temperature during the electrolysis; (iv) evolution of STY during the oxidation, at different applied current densities.

The evolution of tannic acid, intermediates, and COD, is shown in Figure 9a, as determined at 600 A/m^2^, pH 6.5, and selecting a temperature of 20 °C. These curves highlight the efficiency of the electrolysis in removing tannic acid that was first converted into intermediates and then transformed into CO_2_. Figure 9b evidences that a faster COD removal can be obtained by increasing pH, without losses of gaseous chlorine from the cell or formation of chlorate [32,33]. An increase of temperature also determined an increase in the removal rate, as demonstrated in Figure 9c. Finally, in Figure 9d, the effect of current density on the STY is reported. These profiles evidence the role of current in increasing the reactor performance.

**Figure 10:**
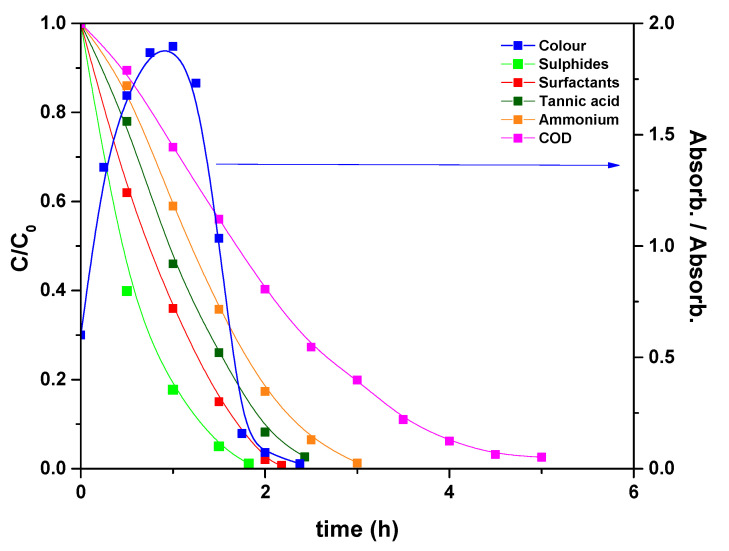
Evolution of the normalized concentrations and colour, expressed as a ratio between the absorbance at time t and the initial absorbance, during the electrolysis of a simulated real wastewater solution: T = 40 °C, pH = 10, applied current density of 600 A/m^2^.

Taking into account these results, electrolysis of a simulated real wastewater solution was performed, using the PVDF HFP_RuO_2_ 60 anode, and applying a current of 600 A/m^2^, at pH 10 and setting temperature at 40 °C [34]; the results are reported in Figure 10. During the electrolysis, surfactants, sulfides, and tannins were oxidized in about 2.5 h, ammonium nitrogen in 3 h, and COD was removed in 5 h. Specifically, the final values, measured as normalized concentrations, were: 0.007 surfactants, 0.011 sulfides, 0.021 tannins, 0.012 ammonium nitrogen and 0.025 COD. Regarding the degradation process, the starting pollutants were first degraded in a large quantity of organic intermediates, mainly constituted of carboxylic acids, which were further oxidized to carbon dioxide [15].

In chloride-containing solutions, since the direct electron-transfer oxidation was negligible [8,35], due to the low oxygen overpotential, chloride removal was due to indirect electrolysis, related to surface redox reactions, generating active chlorine:

2Cl^−^ → Cl_2_ + 2e^−^

C_M_H_N_O_P_ + Cl_2_ → products + 2Cl^−^

Furthermore, sulfide ions were converted into sulfates and ammonium nitrogen in gaseous N_2_. Also the solution colour changed during electrolysis, as evidenced in Figure 10.

**Figure 11:**
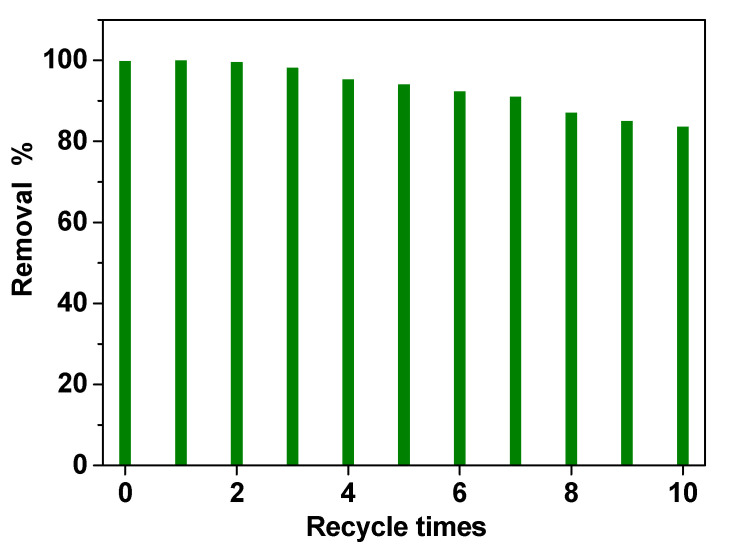
Reusability of the PVDF HFP_RuO_2_ 60 electrode: COD evolution during 10 times treatment.

Finally, recycling experiments were performed to evaluate the stability of the electrode in the simulated real wastewater solution. PVDF HFP_RuO_2_ 60 nanocomposite showed high removal performances even after 10 treatments, as reported in Figure 11, indicating a good stability. A slight decrease in removal efficiency (lower than 15%) in the last three tests (i.e., recycle times 8, 9, and 10) was probably due to carbon accumulation on the electrode surface, during the recycling experiments.

## 4. Conclusions

The morphological and chemical features of the nanocomposite aerogels produced in this work evidenced a RuO_2_ uniform distribution in the porous polymeric matrix, thanks to the fast kinetics in solvents removal during the supercritical CO_2_ drying.

The advantage of using polymeric aerogels, structured from micro- to nanoscale, coupled with active nanomaterials, determined a relevant purification performance on a simulated real wastewater mixture. Specifically, during the electrolysis, surfactants, sulfides, and tannins were oxidized in about 2.5 h, ammonium nitrogen in 3 h, and COD was removed in 5 h. In chloride-containing solutions, the purification was due to indirect electrolysis, related to surface redox reactions generating active chlorine; whereas sulfide ions were converted into sulfates and ammonium nitrogen in gaseous N_2_. Therefore, this work demonstrated that electrochemical oxidation can be performed as the final treatment of tannery wastewaters.
